# Inflammation markers and cognitive performance in breast cancer survivors 20 years after completion of chemotherapy: a cohort study

**DOI:** 10.1186/s13058-018-1062-3

**Published:** 2018-11-15

**Authors:** Kimberly D. van der Willik, Vincent Koppelmans, Michael Hauptmann, Annette Compter, M. Arfan Ikram, Sanne B. Schagen

**Affiliations:** 1 Department of Psychosocial Research and Epidemiology, Netherlands Cancer Institute, Plesmanlaan 121, 1066 CX Amsterdam, the Netherlands; 2 Department of Epidemiology, Erasmus MC - University Medical Center Rotterdam, PO Box 2040, 3000 CA Rotterdam, the Netherlands; 3 Department of Psychiatry, The University of Utah, 501 Chipeta Way, Salt Lake City, UT 84108 USA; 4 Department of Neuro-oncology, Netherlands Cancer Institute, Plesmanlaan 121, 1066 CX Amsterdam, the Netherlands; 5 Brain and Cognition, Department of Psychology, University of Amsterdam, Nieuwe Achtergracht 129-B, 1018 WS Amsterdam, the Netherlands

**Keywords:** Breast cancer, Inflammation, Cognitive performance, Cancer/cancer treatment-related side effects

## Abstract

**Background:**

Inflammation is an important candidate mechanism underlying cancer and cancer treatment-related cognitive impairment. We investigated levels of blood cell–based inflammatory markers in breast cancer survivors on average 20 years after chemotherapy and explored the relation between these markers and global cognitive performance.

**Methods:**

One hundred sixty-six breast cancer survivors who received post-surgical radiotherapy and six cycles of adjuvant cyclophosphamide, methotrexate, and fluorouracil (CMF) chemotherapy on average 20 years before enrollment were compared with 1344 cancer-free women from a population-based sample (50–80 years old). Breast cancer survivors were excluded if they used adjuvant hormonal therapy or if they developed relapse, metastasis, or second primary malignancies. Systemic inflammation status was assessed by the granulocyte-to-lymphocyte ratio (GLR), platelet-to-lymphocyte ratio (PLR), and systemic immune-inflammation index (SII). Cognitive performance was assessed using an extensive neuropsychological test battery from which the general cognitive factor was derived to evaluate global cognitive performance. We examined the association between cancer, the general cognitive factor, and inflammatory markers using linear regression models.

**Results:**

Breast cancer survivors had a lower general cognitive factor than non-exposed participants from the comparator group (mean difference = −0.21; 95% confidence interval (CI) −0.35 to −0.06). Inflammatory markers were higher in cancer survivors compared with non-exposed participants (mean difference for log(GLR) = 0.31; 95% CI 0.24 to 0.37, log(PLR) = 0.14; 95% CI 0.09 to 0.19, log(SII) = 0.31; 95% CI 0.24 to 0.39). The association between higher levels of inflammatory markers and lower general cognitive factor was statistically significant in cancer survivors but not among non-exposed participants. We found a group-by-inflammatory marker interaction; cancer survivors showed additional lower general cognitive factor per standard deviation increase in inflammatory markers (*P* for interaction for GLR = 0.038, PLR = 0.003, and SII = 0.033).

**Conclusions:**

This is the first study to show that (1) cancer survivors have increased levels of inflammation on average 20 years after treatment and (2) these inflammatory levels are associated with lower cognitive performance. Although this association needs verification by a prospective study to determine causality, our findings can stimulate research on the role of inflammation in long-term cognitive problems and possibilities to diminish such problems.

## Background

Patients with cancer frequently report cognitive problems that can affect their quality of life and daily functioning substantially. Studies have shown that patients with non-central nervous system (non-CNS) cancer can experience cognitive problems during and after completion of treatment including chemotherapy, and a subgroup of patients had cognitive problems up to 20 years after treatment [1, 2].

The cancer survivor population is aging and growing because of increased life expectancy and more specifically because of advances in cancer treatment and improved screening. In turn, this results in an increasing number of cancer survivors coping with cognitive problems. The driving forces underlying these cognitive problems have not been sufficiently clarified, impeding the approach and process of developing effective interventions. Cognitive problems in patients with cancer could be induced by cancer itself, cancer-related treatment, or shared risk factors for the development of both cancer and cognitive problems [3, 4]. Disentangling the effects and mechanisms of these causes of disruption of normal cognitive performance is challenging. Different mechanisms, including genetic susceptibility, telomere shortening, changes in hormone levels, and inflammation, have been proposed and revealed [3].

In recent years, inflammation in particular has been suggested as an important and potentially intervenable mechanism in the pathogenesis of cognitive problems in patients with cancer. Higher levels of inflammatory factors such as cytokines are observed in patients with cancer prior to start of any treatment [5], during chemotherapy [6–10], and after chemotherapy [11, 12] up to 5 years after treatment initiation [13]. Several studies found an association between cytokines and cognitive impairment in patients with cancer across different cognitive domains, such as psychomotor speed [8], executive functioning [14], and memory [5, 10, 11, 13]. However, these studies did not agree on the involved cytokines or on the affected cognitive domain. Moreover, because the longest follow-up in these studies was 5 years, it remains unknown whether inflammation also has a role in longer-term or late cognitive problems. Filling this knowledge gap is important as insight into underlying causes of (long-term) cognitive impairment helps to identify those cancer patients at increased risk of developing cognitive problems and opens venues for preventive and therapeutic interventions.

Most studies examined the inflammation status by investigating cytokines using different cytokine panels [5, 6, 8–19]. In contemporary studies, systemic inflammatory response markers measured in blood, including the neutrophil-to-lymphocyte ratio (NLR), platelet-to-lymphocyte ratio (PLR), and systemic immune-inflammation index (SII), are increasingly used. These markers have reliable prognostic and predictive value in patients with cancer and can easily be calculated from readily available standard full blood examination, making them more convenient to use in a clinical setting [20–24]. If related to cognitive problems, these markers could potentially be used as biomarkers for cancer-related cognitive impairment.

In this study, we investigated global cognitive performance, levels of blood cell–based inflammatory markers, and their relation in breast cancer survivors who had received post-surgical radiotherapy and six cycles of adjuvant cyclophosphamide, methotrexate, and fluorouracil (CMF) chemotherapy on average more than 20 years previously. We furthermore examined whether inflammation and cognitive performance were differentially associated between breast cancer survivors and cancer-free women from a population-based sample.

## Methods

### Study population

In this study, we selected women who had survived breast cancer and had received adjuvant CMF chemotherapy. We compared them with women from the general population, who were cancer-free and had never received chemotherapy.

### Breast cancer survivors

Women with a history of unilateral, invasive breast cancer were identified on the basis of registries of the Netherlands Cancer Institute in Amsterdam and the Daniel den Hoed Cancer Clinic of the Erasmus Medical Center in Rotterdam as described previously [2]. Briefly, women were selected if they had received post-surgical radiotherapy and six cycles of adjuvant CMF chemotherapy between 1976 and 1995.

Breast cancer survivors were eligible if they were 50–80 years old at the time of inclusion in 2008, if invasive breast cancer was their first and only malignancy, if they had not developed relapse or distant metastasis, if they had sufficient command of the Dutch language, and if they did not have any contraindications for magnetic resonance imaging (MRI). In addition, ever use of hormonal therapy was applied as an exclusion criterion. Because adjuvant hormonal therapy was not part of the standard treatment for patients with breast cancer in the Netherlands until the mid-1990s, only a few women received this treatment. To enhance homogeneity within the group of breast cancer survivors, we included hormone treatment-naïve cancer survivors only.

**Fig. 1:**
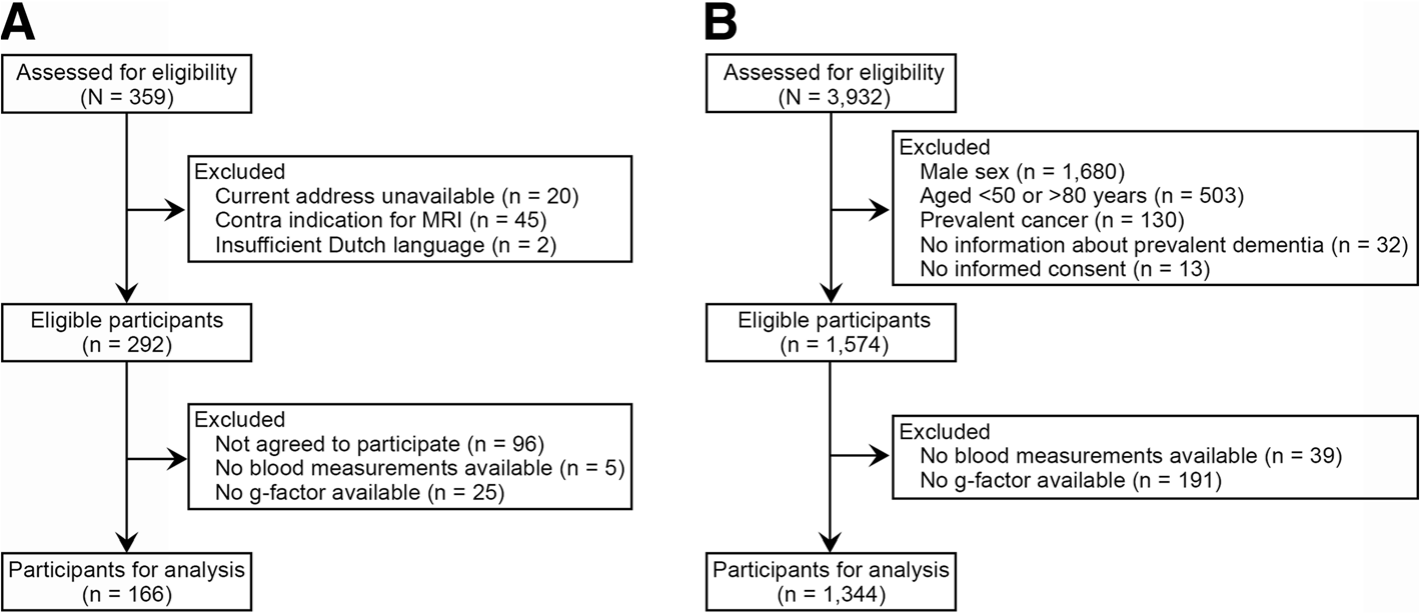
**a** Flowchart for breast cancer survivors. **b** Flowchart for non-exposed participants. Abbreviation: *MRI* magnetic resonance imaging

Three hundred fifty-nine breast cancer survivors were assessed for eligibility and 292 were selected. Of these 292 women, 196 agreed to participate and provided informed consent. We previously reported on cognitive performance of these survivors in comparison with cancer-free women identified within the Rotterdam Study [2]. For the present study, the following additional inclusion criteria were defined: availability of blood measurements and completeness of neuropsychological test data to calculate the general cognitive factor. Thirty of the 196 (15.3%) breast cancer survivors were excluded because of missing data on blood measurements (*n* = 5) and incomplete data of neuropsychological tests (*n* = 25, Fig. 1a). Because breast cancer survivors did not receive an extensive dementia screening, history of dementia was not applied as an exclusion criterion. However, based on the interviews with a trained psychologist, subjective memory complaints, cognitive tests, and brain MRI, it is unlikely that the included breast cancer survivors had dementia at the time of examinations.

### Population-based non-exposed participants

Cancer-free women were selected from the Rotterdam Study, an ongoing population-based prospective cohort that started in 1990 in Rotterdam, the Netherlands. The main objective of the Rotterdam Study is to investigate risk factors of diseases in the elderly. By the end of 2008, the Rotterdam Study consisted of three subcohorts, comprising 14,926 individuals. The design of the Rotterdam Study was described in detail previously [25].

The third subcohort (RS-III) started in 2006 and was the first cohort in which an extensive set of neuropsychological tests was implemented at baseline. For this reason, RS-III was chosen as the reference subcohort, which was composed of 3392 participants (65% out of invitees). From these participants, women 50–80 years old without a history of cancer or dementia were eligible as non-exposed participants (*n* = 1574). This sample comprised the non-exposed participants used in our previous cognitive study [2]. Two hundred thirty persons were additionally excluded because of lack of blood measurements (*n* = 39) and incomplete data of neuropsychological tests (*n* = 191), resulting in 1344 non-exposed participants (Fig. 1b).

### Assessment of inflammatory markers

All participants had fasting blood samples taken during the research center visit. Full blood count measurements were performed by using a COULTER^®^ Ac·T diff2™ Hematology Analyzer (Beckman Coulter, San Diego, CA, USA) directly after the blood sample was drawn. Hematologic measurements included absolute granulocyte, lymphocyte, and platelet counts in 10^9^ per liter.

We used the granulocyte count as proxy for the neutrophil count because we did not have this measurement available in our sample. Because most of the granulocytes are represented by neutrophils, we believe this did not affect our results [26, 27]. For accuracy purposes, we will refer to the granulocyte-to-lymphocyte ratio (GLR) instead of using the term NLR.

The GLR and PLR were calculated as the ratio of granulocyte count to lymphocyte count and as the ratio of platelet count to lymphocyte count, respectively [28]. The SII was defined as platelet count times the GLR [22]. Because they are either ratios or indices, the derived inflammatory markers did not have a unit.

### Assessment of cognitive performance

Cognitive performance was evaluated between November 2009 and June 2010 for breast cancer survivors and between February 2006 and December 2008 for non-exposed participants on the same day the blood sample was drawn. Cognitive performance was assessed by a neuropsychological battery in the research center of the Rotterdam Study. Six tests were administered: the Mini–Mental State Examination, Letter-Digit Substitution Test (LDST), Word Fluency Test (WFT), Stroop Test (reading, naming, and interference), Purdue Pegboard Test (PPB) (right, left, and both hands), and 15-Word Learning Test (15-WLT) (immediate recall, delayed recall, and recognition). Global cognitive performance was assessed via the general cognitive factor, which was generated by using principal component analysis of the following tests: LDST (total completion time), WFT (number of words), Stroop interference (time in seconds, adjusted for errors), PPB test (total number of pins across three subtasks), and 15-WLT (number of words during delayed recall) [29].

### Other assessments

We assessed education level (primary: primary education; lower: lower general education, intermediate general education, or lower vocational education; intermediate: intermediate vocational education or higher general education; higher: higher vocational education or university) and smoking status (current, former, or never) by interview. Body mass index (BMI) (in kilograms per square meter) was computed from measurements of height and weight. Diabetes mellitus was defined as use of antidiabetic medication, a fasting serum glucose level of at least 7.1 mmol/L, or a random serum glucose level of at least 11.1 mmol/L [30]. History of stroke or myocardial infarction was assessed by interview [31, 32]. Symptoms of depression were evaluated with the Center for Epidemiologic Studies Depression scale (CES-D), which was converted to a sum-score [33]. We had no information about anxiety and fatigue and therefore could not control for these symptoms.

### Statistical analyses

Linear regression models were used to investigate mean differences in the general cognitive factor and inflammatory markers between breast cancer survivors and non-exposed participants. Inflammatory markers were log-transformed because of their skewed distribution. We constructed two nested models: model I was adjusted for age (continuous) and education (four categories), and model II was additionally adjusted for smoking status (three categories), BMI (continuous), diabetes mellitus (yes/no), history of stroke (yes/no), history of myocardial infarction (yes/no), and CES-D sum-score (continuous). To investigate whether levels of the general cognitive factor were explained by different inflammatory markers, we adjusted additionally for each inflammatory marker separately.

The association between the general cognitive factor and inflammatory markers was investigated for breast cancer survivors and non-exposed participants using linear regression models. To study whether this association was stronger in breast cancer survivors than in non-exposed participants, we computed interaction terms between history of cancer/cancer treatment and each inflammatory marker. We explored effect modification by stratifying for mean BMI.

Since mean age was higher in the breast cancer survivors compared with the non-exposed participants (Table 1), we repeated all analyses using age-matched non-exposed participants to minimize residual confounding. These analyses provided estimates comparable to the analyses using all non-exposed participants and therefore are not reported separately.

**Table 1:** Demographics and characteristics of breast cancer survivors and non-exposed participants

Characteristic	Breast cancer survivors (*n* = 166)	Non-exposed participants (*n* = 1344)	*P*
Age in years, mean (SD)	64.0 (6.7)	57.9 (5.2)	<0.001
Education level, no. (%)			<0.001
Primary	14 (8.4)	158 (11.8)	
Low	59 (35.5)	616 (45.8)	
Intermediate	33 (19.9)	287 (21.4)	
High	60 (36.1)	283 (21.1)	
Body mass index in kg/m^2^, mean (SD)	26.9 (4.6)	27.4 (4.8)	0.181
Smoking status, no. (%)			<0.001
Current	16 (9.6)	295 (21.9)	
Former	93 (56.0)	574 (42.7)	
Diabetes mellitus, no. (%)	14 (8.4)	54 (4.0)	0.008
History of stroke, no. (%)	1 (0.6)	19 (1.4)	0.715
History of myocardial infarction, no. (%)	6 (3.6)	11 (0.8)	0.001
CES-D sum-score, mean (SD)	14.5 (3.6)	14.8 (4.4)	0.450
General cognitive factor, mean (SD)	−0.39 (1.14)	0.05 (0.97)	<0.001
Inflammatory markers, median (IQR)			
GLR	2.06 (1.67–2.66)	1.52 (1.20–1.92)	<0.001
PLR	145 (119–176)	124 (102–151)	<0.001
SII	618 (469–796)	443 (328–595)	<0.001
Age at breast cancer diagnosis in years, mean (SD)	42.9 (5.6)		
Time since breast cancer diagnosis, mean (SD)	21.0 (4.5)		

Abbreviations: *CES-D* Center for Epidemiologic Studies Depression Scale, *GLR* granulocyte-to-lymphocyte ratio, *IQR* interquartile range, *PLR* platelet-to-lymphocyte ratio, *SD* standard deviation, *SII* systemic immune-inflammation index

Multiple imputation was used for missing data on covariates (generally between 0.07% and 0.3% with a maximum of 1.8% for the CES-D sum-score) with five imputed datasets, based on history of cancer/cancer treatment, inflammatory markers, general cognitive factor, and other covariates (that is, age, sex, education, BMI, smoking status, presence of diabetes mellitus, history of stroke, history of myocardial infarction, and CES-D sum-score). Rubin’s method was used for pooled regression coefficients (β) and 95% confidence intervals (CIs) [34]. All analyses were performed by using IBM SPSS Statistics Version 24.0 and RStudio Version 3.3.2. All statistical tests were two-sided, and a *P* value of less than 0.05 was considered statistically significant.

## Results

Characteristics of breast cancer survivors and non-exposed participants are presented in Table 1. Breast cancer survivors were older than non-exposed participants. Additionally, they generally had completed higher levels of education and more often had diabetes mellitus and a history of myocardial infarction. Lastly, although the numbers of never smokers were similar between the two groups, breast cancer survivors were more frequently former smokers and less often current smokers.

### Inflammatory markers

Breast cancer survivors had higher median levels of GLR, PLR, and SII than non-exposed participants. History of breast cancer/cancer treatment was associated with higher inflammatory markers, also after adjustment for age, education, smoking, BMI, diabetes mellitus, history of stroke, history of myocardial infarction, and CES-D sum-score (mean difference for log(GLR) = 0.31, 95% CI 0.24 to 0.37, log(PLR) = 0.14, 95% CI 0.09 to 0.19, log(SII) = 0.31, 95% CI 0.24 to 0.39; Table 2). Inflammatory markers were positively associated with age in both groups [35].

**Table 2:** Association between the general cognitive factor and history of cancer and inflammatory markers and history of cancer

Outcome	Model I	Model II
	Mean difference (95% CI)	Mean difference (95% CI)
Inflammatory marker*		
Log GLR	0.30 (0.24 to 0.36)	0.31 (0.24 to 0.37)
Log PLR	0.16 (0.10 to 0.21)	0.14 (0.09 to 0.19)
Log SII	0.30 (0.23 to 0.38)	0.31 (0.24 to 0.39)
Cognition^†^		
General cognitive factor	−0.18 (−0.34 to −0.03)	−0.21 (−0.35 to −0.06)

Abbreviations: *CI* confidence interval, *GLR* granulocyte-to-lymphocyte ratio, *PLR* platelet-to-lymphocyte ratio, *SII* systemic immune-inflammation index

Model I is a linear regression of the general cognitive factor or log-transformed inflammatory markers on cancer status adjusted for age and education. Model II is as model I plus adjustment for smoking status, body mass index, diabetes mellitus, history of stroke, history of myocardial infarction, and Center for Epidemiologic Studies Depression Scale (CES-D) sum-score

*Mean difference in general cognitive factor between breast cancer survivors and non-exposed participants

^†^Mean difference in inflammatory markers between breast cancer survivors and non-exposed participants

### Cognitive performance

Breast cancer survivors had a lower general cognitive factor than non-exposed participants (mean difference = −0.21, 95% CI −0.35 to −0.06, corresponding with an effect of 3.6 years of age given a decline in general cognitive factor of 0.59 points per 10 years; Table 2) [29]. Further adjustment for inflammatory factors changed the estimates slightly, indicating that inflammatory markers explained only a small part of the difference in general cognitive factor in addition to the effect of history of cancer/cancer treatment (mean difference for history of cancer/cancer treatment after adjustment for log(GLR) = −0.18; 95% CI −0.33 to 0.02, log(PLR) = −0.21; 95% CI −0.36 to 0.06, log(SII) = −0.19; 95% CI −0.34 to 0.03).

### Association between cognitive performance and inflammatory markers by cancer status

A lower general cognitive factor was associated with higher inflammatory markers in breast cancer survivors (Table 3). In non-exposed participants, higher inflammatory markers tended to be associated with a lower general cognitive factor, albeit not statistically significant.

**Table 3:** Association between the general cognitive factor and inflammatory markers in breast cancer survivors and in non-exposed participants

Inflammatory marker per SD increase	Breast cancer survivors	Non-exposed participants	*P* for interaction^†^
	Mean difference* (95% CI)	Mean difference* (95% CI)	
Model I			
Log GLR	−0.24 (−0.40 to −0.08)	−0.04 (−0.09 to 0.00)	0.061
Log PLR	−0.13 (−0.29 to 0.03)	0.05 (0.01 to 0.10)	0.003
Log SII	−0.22 (−0.38 to −0.07)	−0.03 (−0.08 to 0.01)	0.053
Model II			
Log GLR	−0.23 (−0.39 to −0.08)	−0.02 (−0.07 to 0.02)	0.038
Log PLR	−0.18 (−0.33 to −0.02)	0.03 (−0.01 to 0.08)	0.003
Log SII	−0.23 (−0.38 to −0.07)	−0.01 (−0.06 to 0.03)	0.033

Abbreviations: *CI* confidence interval, *GLR* granulocyte-to-lymphocyte ratio, *PLR* platelet-to-lymphocyte ratio, *SD* standard deviation, *SII* systemic immune-inflammation index

Model I is a linear regression of the general cognitive factor on each log-transformed inflammatory marker adjusted for age and education. Model II is as model I plus adjustment for smoking status, body mass index, diabetes mellitus, history of stroke, history of myocardial infarction, and Center for Epidemiologic Studies Depression Scale (CES-D) sum-score

*Mean difference in general cognitive factor per standard deviation increase in inflammatory marker

^†^*P* value for interaction term between history of cancer/cancer treatment and inflammatory marker

**Fig. 2:**
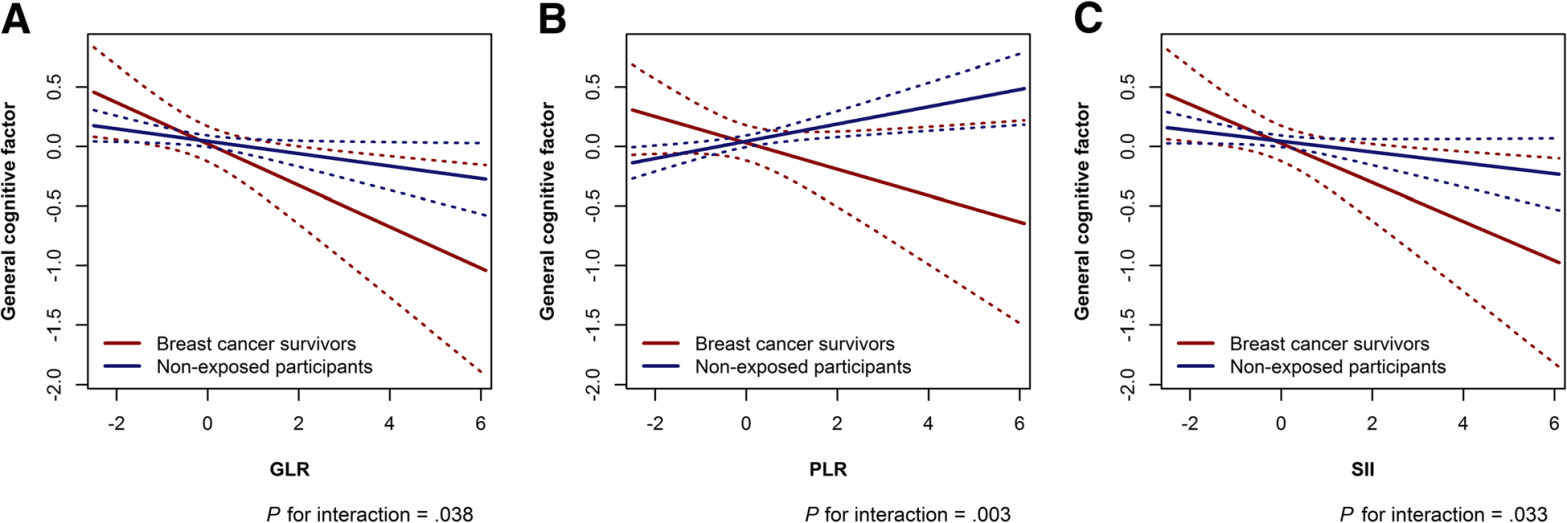
**a** Interaction of log(GLR) and cancer status with the general cognitive factor as outcome. **b** Same as a, for log (PLR). **c** Same as a and b, for log(SII). Model used for figure is adjusted for age only. Abbreviations: *GLR* granulocyte-to-lymphocyte ratio, *PLR* platelet-to-lymphocyte ratio, *SII* systemic immune-inflammation index

The interaction term between inflammatory markers and history of cancer/cancer treatment was significant for each inflammatory marker, indicating that the association between higher inflammation levels and lower general cognitive factor was more pronounced in breast cancer survivors than in non-exposed participants (*P* for interaction between cancer and standardized log-transformed GLR = 0.038, PLR = 0.003, and SII = 0.033; Fig. 2).

The association between higher inflammatory markers and lower general cognitive factor differed more between breast cancer survivors and non-exposed participants with a higher BMI than in those with a lower BMI. However, stratified analyses for BMI showed that the effect of one–standard deviation increase in inflammatory marker on general cognitive factor was higher among breast cancer survivors with a BMI below 27.3 kg/m^2^ compared with those with a higher BMI (Table 4).

**Table 4:** Association between the general cognitive factor and inflammatory markers in breast cancer survivors and in non-exposed participants stratified for mean body mass index

Inflammatory marker per SD increase	Breast cancer survivors		Non-exposed participants		*P* for interaction^†^
	Mean difference*	95% CI	Mean difference*	95% CI	
BMI < 27.3 kg/m^2^					
	*n* = 104		*n* = 749		
Log GLR	−0.29	−0.49 to −0.10	−0.04	−0.10 to 0.02	0.480
Log PLR	−0.22	−0.42 to −0.02	0.01	−0.05 to 0.08	0.309
Log SII	−0.28	−0.48 to −0.09	−0.04	−0.10 to 0.02	0.564
BMI > 27.3 kg/m^2^					
	*n* = 62		*n* = 595		
Log GLR	−0.16	−0.41 to 0.09	0.01	−0.06 to 0.08	0.013
Log PLR	−0.16	−0.42 to 0.10	0.05	−0.02 to 0.12	<0.001
Log SII	−0.12	−0.38 to 0.14	0.02	−0.05 to 0.09	0.005

Abbreviations: *BMI* body mass index, *CI* confidence interval, *GLR* granulocyte-to-lymphocyte ratio, *PLR* platelet-to-lymphocyte ratio, *SD* standard deviation, *SII* systemic immune-inflammation index

Model I is a linear regression of the general cognitive factor on each log-transformed inflammatory marker adjusted for age and education. Model II is as model I plus adjustment for smoking status, diabetes mellitus, history of stroke, history of myocardial infarction, and Center for Epidemiologic Studies Depression Scale (CES-D) sum-score.

*Mean difference in general cognitive factor per standard deviation increase in inflammatory marker

^†^*P* value for interaction term between history of cancer/cancer treatment and inflammatory marker

## Discussion

This study is the first report investigating the association between blood cell–based inflammatory markers and cognitive performance in breast cancer survivors with an average time since cessation of chemotherapy of more than 20 years. Breast cancer survivors had lower global cognitive performance and higher inflammatory markers compared with women without a history of cancer. The tendency for lower global cognitive performance with higher inflammatory markers was more pronounced in breast cancer survivors, suggesting a potential role for inflammation in the pathophysiology of cognitive problems in cancer survivors. This effect was not modified by BMI. More insight in mechanisms underlying cognitive problems could help identifying those women who are at an increased risk of cognitive problems and developing prevention strategies.

We previously reported on differences in cognitive performance between breast cancer survivors and non-exposed participants [2]. In this previous study, we tested between-group performance differences of individual cognitive outcome measures that were currently used to construct the general cognitive factor and observed that breast cancer survivors performed worse compared with non-exposed participants within several cognitive domains. This suggested that cognitive problems in cancer survivors can be long-lasting. In the present study, we evaluated global cognitive performance using the general cognitive factor because we did not expect a specific cognitive domain to be affected by inflammation. We chose to use a robust cognitive summary measure, thereby reducing the number of comparisons.

Interestingly, levels of inflammatory markers were higher in breast cancer survivors, compared with non-exposed participants, on average 20 years after cancer treatment. Inflammation plays a critical role in tumorigenesis, tumor progression, and cancer metastasis [36, 37]. Research has shown that chronic inflammation is associated with an increased cancer risk [37]. Moreover, different markers of inflammation, such as cytokines, C-reactive protein, and NLR, are often elevated in patients with cancer and are associated with poor survival [9, 15–17, 24, 38]. One study investigating inflammation levels after cancer treatment found that C-reactive protein and cytokine levels were elevated up to 5 years after treatment [19]. Our observation that systemic inflammation markers are higher in breast cancer survivors compared with non-exposed participants on average 20 years after cancer treatment suggests deregulation of the immune system. Whether this is a consequence of cancer or cancer treatment (or both) or a pre-existing deregulation before cancer development cannot be determined with the present study.

The found association of blood cell–based inflammatory markers and cognitive performance in breast cancer survivors is in line with previous observations before, during, and shortly after therapy [6, 17, 18]. Two studies investigated the link between inflammation and cognitive performance prior to the start of cancer treatment. The first study showed that elevated levels of interleukin-6 (IL-6) in patients with acute myelogenous leukemia or myelodysplastic syndrome were associated with poorer executive functioning before cancer treatment [14]. The second study showed that high levels of soluble tumor necrosis factor receptor type II (sTNF-RII) were related to reduced verbal memory performance in patients with newly diagnosed breast cancer [5]. More studies in patients with breast cancer have tried to elucidate the role of inflammation in impaired cognitive performance during chemotherapy and two of these studies identified specific cytokines to be involved. Williams et al. focused on sTNR-RII and found that higher levels of this receptor were associated with visual memory performance [10]. Cheung et al. observed an association between increased levels of IL-6 and IL-1β and poorer psychomotor speed performance during chemotherapy [8]. Shortly after cancer treatment, higher levels of sTNF-RII were associated with increased memory complaints [11], and on average 5 years after cancer treatment, elevated IL-6 and TNFα levels were associated with worse verbal memory [13]. Importantly, the association between inflammation and cognitive performance is supported by animal studies. Acute peripheral immune challenges using lipopolysaccharide resulted in cognitive impairments in a spatial working memory task in mice. Cognitive impairments were observed 1.5–2 h after injection in tumor-bearing mice but not in tumor-free mice. These cognitive effects could be prevented when using a technique to enhance innate immune reactivity [39]. Together, these results support the hypothesis that inflammation has a role in the complex pathogenesis of both short-term and longer-term cognitive problems in patients with cancer.

Owing to our study design, we cannot determine whether the association between inflammation and impaired cognitive performance is causal. However, also a causal association could not illuminate the exact underlying mechanisms by which inflammation leads to brain changes and subsequent cognitive problems. Peripheral pro-inflammatory cytokines are able to cross the blood–brain barrier, which may initiate the release of local cytokines [40]. Local cytokine production could result in neurotransmitter deregulation, increased oxidative stress, and decreased neurogenesis and neuroplasticity, which in turn can lead to cognitive dysfunction [41]. It is also possible that inflammation induces epigenetic changes and chromosomal instability, which can be persistent and therefore could be associated with long-term cognitive problems [42].

Our study has several strengths. First, we have a large sample size of breast cancer survivors who have been treated on average more than 20 years ago, enabling us to investigate long-term effects. Moreover, we used non-exposed participants from a population-based cohort study, who underwent the same examinations as the breast cancer survivors. This design provided standardized ascertainments of outcome and covariates. All participants received a neuropsychological test battery, enabling us to investigate global cognitive function by the general cognitive factor. Lastly, we were able to investigate inflammation status using blood cell–based inflammatory markers, which are low-cost and easy to use in the clinic.

Study limitations include the design by which we cannot disentangle the effects of cancer and cancer treatment on cognition and levels of inflammatory markers. Some studies show that patients treated with chemotherapy have higher inflammatory markers during and after treatment compared with chemotherapy-naïve patients [12]. However, because inflammatory markers and cognitive problems can already occur in patients with newly diagnosed cancer, it is unlikely that inflammation is important only in chemotherapy-treated patients [5]. Owing to the cross-sectional design, we do not have information about cognitive performance and levels of inflammatory markers before cancer diagnosis and treatment. Moreover, patients with breast cancer nowadays receive chemotherapy regimens other than CMF, either with or without adjuvant endocrine therapy, limiting the generalizability to current patients with breast cancer. However, cyclophosphamide and 5-fluoroacil are still frequently used in other regimens for adjuvant chemotherapy. Furthermore, we were not able to exclude individuals whose systemic inflammatory markers may have been elevated because of acute infections and to control for acute-phase reactants such as C-reactive protein, but we expect that this effect is similar for cancer survivors and non-exposed participants. Lastly, we need to emphasize that by measuring the GLR, PLR, and SII, we cannot identify the exact phenotype of the underlying immune cell populations. Although these markers are proven to be related to chronic systemic inflammation, it is unknown whether they also reflect higher levels of pro-inflammatory cytokines. In other words, we cannot confirm that observed shifts in the granulocytes, lymphocytes, and platelets cause higher cytokine levels and thereby are functional. To elucidate the exact immune cell populations involved in increases of the GLR, PLR, and SII, determination of different cytokines is needed.

## Conclusions

We found that breast cancer survivors who had been treated with chemotherapy on average more than 20 years ago have higher blood cell–based inflammatory markers compared with women without a history of cancer. Higher levels of inflammatory markers tended to be associated with poorer cognitive performance in both cancer survivors and cancer-free women, and expression was stronger in breast cancer survivors. This finding suggests that inflammation could have a role in the pathogenesis of long-term cognitive impairment in cancer survivors. Further prospective studies are important to determine the causality of the association and to investigate the effects of lowering inflammation on the development of cognitive problems in cancer patients and survivors, for instance, by exercise or anti-inflammatory drugs.

## Abbreviations

15-WLT: 15-Word Learning Test
BMI: Body mass index
CES-D: Center for Epidemiologic Studies Depression Scale
CI: Confidence interval
CMF: Cyclophosphamide, methotrexate, and fluorouracil
GLR: Granulocyte-to-lymphocyte ratio
IL: Interleukin
LDST: Letter-Digit Substitution Test
MRI: Magnetic resonance imaging
NLR: Neutrophil-to-lymphocyte ratio
PLR: Platelet-to-lymphocyte ratio
PPB: Purdue Pegboard Test
RS-III: Third subcohort of the Rotterdam Study
SII: Systemic immune-inflammation index
sTNF-RII: Soluble tumor necrosis factor receptor type II
TNF: Tumor necrosis factor
WFT: Word Fluency Test
